# Supplementary material for: Assessment of Physicochemical and In Vivo Biological Properties of Polymeric Nanocapsules Based on Chitosan and Poly(N-vinyl pyrrolidone-alt-itaconic anhydride)
Source: Polymers (Basel). 2022 Apr 28;14(9):1811. doi: 10.3390/polym14091811 (PMC9104533; doi:10.3390/polym14091811)
Supplement: Supplementary file 1 [file polymers-14-01811-s001.zip › polymers-1622605-supplementary.pdf]

Supporting Information

Assessment of physicochemical and *in vivo* biological properties of polymeric nanocapsules based on chitosan and poly(*N*-vinyl pyrrolidone-*alt*-itaconic anhydride)

Kheira Zanoune Dellali[1], Mohammed Dellali[1], Delia Mihaela Rata[2*], Anca Niculina Cadinoiu[2], Leonard Ionut Atanase[2*], Marcel Popa[2,3*], Mihaela-Claudia Spataru[4], Carmen Solcan[4]

[1]Faculty of Technology, University Hassiba Benbouali, BP 151 02000, Chlef, Algeria

[2]"Apollonia" University of Iasi, Pacurari Street, No. 11, 700511, Iasi, Romania

[3]Academy of Romanian Scientists, Splaiul Independentei Street, No 54, 050094, Bucharest, Romania

[4]"Ion Ionescu de la Brad" University of Life Sciences, Mihail Sadoveanu Alley, No 8, 700489, Iasi, Romania

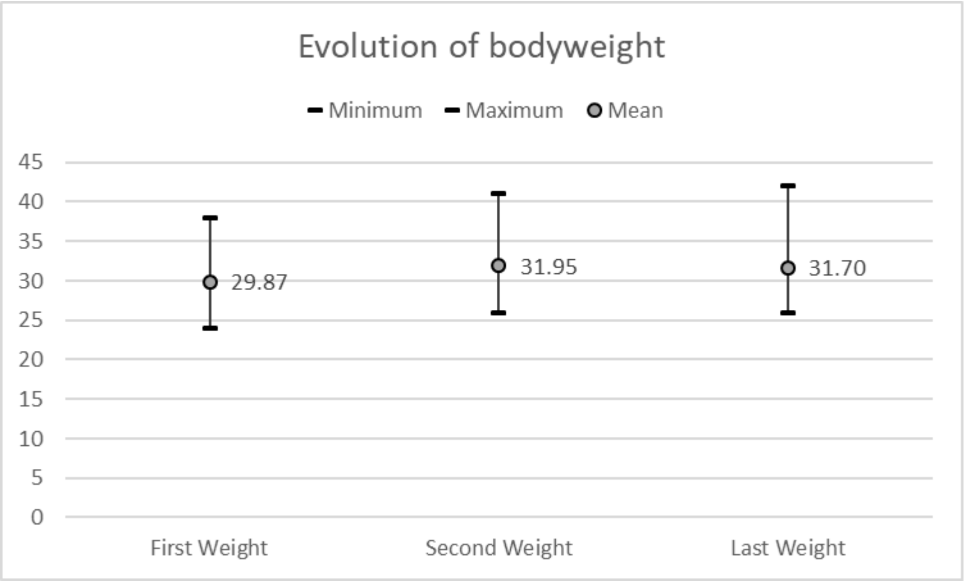

Figure S1: Evolution of mice bodyweight through the experiment (the first measurement was made in the first day of the experiment, the second one at 10 day and the last one at the final of the experiment)

Table S1. *p*-values of Student's t-test for simple 5-FU compared with 5-FU loaded NCs

***p*-value**	**CN-1-5FU**	**CN-2-5FU**	**CN-3-5FU**	**CN-4-5FU**	**CN-5-5FU**	**CN-6-5FU**	**CN-7-5FU**
**5-FU**	0.0217	0.0350	0.0308	0.0304	0.0394	0.0454	0.0238

The statistical significance of drug release kinetics was analyzed by the Student's t-test. The values are expressed as mean ± SE of three parallel measurements, $p<0.05$ being considered significant.
